# Contribution of the *TCF7L2* rs7903146 (C/T) gene polymorphism to the susceptibility to type 2 diabetes mellitus in Cameroon

**DOI:** 10.1186/s40200-015-0148-z

**Published:** 2015-04-14

**Authors:** Magellan Guewo-Fokeng, Eugene Sobngwi, Barbara Atogho-Tiedeu, Olivier Sontsa Donfack, Jean Jacques N Noubiap, Elvis Ndonwi Ngwa, Edith Pascale Mato-Mofo, Priscille Pokam Fosso, Eric Djahmeni, Rosine Djokam-Dadjeu, Marie-Solange Evehe, Folefac Aminkeng, Wilfred F Mbacham, Jean Claude Mbanya

**Affiliations:** Department of Biochemistry, Faculty of Science, University of Yaoundé I, Yaoundé, Cameroon; Laboratory for Molecular Medicine and Metabolism, Biotechnology Center, University of Yaoundé I, Yaoundé, Cameroon; Department of Internal Medicine and Specialties, Faculty of Medicine and Biomedical Sciences, University of Yaoundé I, Yaoundé, Cameroon; National Obesity Center, Yaoundé Central Hospital, University of Yaoundé 1, Yaoundé, Cameroon; Department of Medicine, Groote Schuur Hospital and University of Cape Town, Cape Town, South Africa; Medical Diagnostic Center, Yaoundé, Cameroon; The Canadian Pharmacogenomics Network for Drug Safety (CPNDS), Center for Molecular Medicine and Therapeutics, Department of Medical Genetics, University of British Columbia, Vancouver, Canada; Laboratory for Public Health Research Biotechnologies, Biotechnology Center, University of Yaoundé I, Yaoundé, Cameroon

**Keywords:** Type 2 diabetes, Genetic association, Transcription factor 7-like 2 (*TCF7L2*), Sub-Saharan Africa, Cameroon

## Abstract

**Background:**

Data on the genetic variants for type 2 diabetes mellitus (T2DM) in sub-Saharan African populations are very scarce. This study aimed to investigate the association of transcription factor 7-like (TCF7L2) with T2DM in a Cameroonian population and explore possible genotype-phenotype correlation.

**Methods:**

This is a case–control study involving 37 T2DM patients and 37 non-diabetic volunteers of Cameroonian ethnicity aged 40 years old and above. We collected clinical and biological data to determine phenotypic traits. *TCF7L2* was analyzed by genotyping for rs7903146 (C/T) using PCR-RFLP. Biochemical analyses were performed using a spectrophotometer with Chronolab kits. Statistical analyses were carried out using IBM SPSS, PS and Quanto.

**Results:**

*TCF7L2* was associated with T2DM in this Cameroonian population (*p* = 0.013 for alleles, and *p* = 0.013 for genotypes). The risk allele was C (9.5% patients vs. 0% healthy controls, OR = 16.56) and the protective allele was *T* (90.5% patients vs. 100.0% healthy controls, OR = 0.06). The risk genotype was C/T (18.9% patients vs. 0% healthy controls, OR = 18.44), while the protective genotype was T/T (81.1% patients vs. 100.0% healthy controls, OR = 0.054). The statistical power was 99.99%. *TCF7L2* was not preferentially associated with a specific disease phenotype.

**Conclusion:**

*TCF7L2* is associated with T2DM in this Cameroonian population. The association is not dependent on a specific T2DM phenotype. Clinical genetic testing for *TCF7L2* can help to predict the occurrence of T2DM in Cameroon.

## Background

Type 2 diabetes mellitus (T2DM) is a metabolic disease characterized by hyperglycemia that can occur as a result of impaired insulin secretion, insulin resistance in peripheral tissues and increased hepatic glucose output [1]. T2DM causes significant morbidity, disability and early mortality, and imposes a huge economic burden on the individual, national healthcare and economy [2,3]. T2DM has reached epidemic proportions worldwide. The International Diabetes Federation (IDF) estimates the number of adults with diabetes in the world at 381.8 million in 2013, a number that will expand to 591.9 million in 2035. The increase of the prevalence of diabetes will be highest in sub-Saharan Africa, with a projected growth of 109.6%, from 19.8 million in 2013 to 41.5 million in 2035 [3].

T2DM involves a complex interaction between genetic and environmental factors such as dietary habits and lifestyle. It is often associated with a strong genetic predisposition but the genetics of this form of diabetes remains complex and not well understood [4]. With the advent of high throughput genome-wide association studies, several susceptibility loci for T2DM have been identified [5], including the transcription factor 7-like 2 (*TCF7L2*) which is involved in insulin secretion [6,7]. Following the initial publication of an association of common variants in *TCF7L2* with T2DM in people of European descent [8], an avalanche of replication reports has confirmed this finding and demonstrated that this genetic association is highly reproducible in various ethnic populations [9-20]. A large Human Genome Epidemiology (HuGE) review and meta-analysis has suggested that *TCF7L2* is involved in near 1/5 of all T2DM [21].

Africans harbour an increased susceptibility in the incidence of T2DM and the occurrence of complications [22]. However, the genetic basis of T2DM in native African populations has been understudied. The determination of genetic risk factors of T2DM in sub-Saharan African populations that will face the largest increase of the prevalence of diabetes in the next decades is highly desirable. Such data might improve the prediction, prevention, stratification, disease management and enhance the understanding of the heterogeneity and pathophysiology of T2DM in sub-Saharan African populations.

The association of *TCF7L2* with T2DM has not been studied in Cameroon till date. The aim of this study was to evaluate the association of the *TCF7L2* rs7903146 (C/T) gene polymorphism with T2DM in a Cameroonian population and explore the correlation with phenotypic traits (genotype-phenotype correlation).

## Methods

### Ethics statement

Ethical clearance was obtained from the National Ethical Review Board of the Cameroon Ministry of Public Health. Written informed consent was obtained from all the participants. The study was conducted in accordance with the Helsinki Declaration.

### Study population

This is a case–control study involving 37 T2DM patients and 37 non-diabetic volunteers of Cameroonian ethnicity aged 40 years old and above. T2DM patients, diagnosed according to the IDF criteria [23], were consecutively recruited through the outpatient clinic of the National Obesity Center of the Yaoundé Central Hospital. Non-diabetic controls were recruited from the general population and included in the study after being tested negative for diabetes.

For all participants, we collected data on the sex and age; we measured height, waist and hip circumference to the nearest 0.5 cm, and weight in light clothes to the nearest 0.1 kg, and we then calculated the body mass index (BMI) as weight in kg/height^2^ in m^2^, and the waist-to-hip ratio. Blood pressure was reported as the mean of two measurements performed at least three minutes apart, in the right arm with the subject sited after a 15-min rest with an automatic sphygmomanometer Omron HEM-705 CP (Omron Corporation, Tokyo, Japan). We collected blood samples for biochemical and molecular assays.

### Biochemical assays

Fasting plasma glucose (glucose oxidase–peroxidase method), serum cholesterol (cholesterol oxidase phenol4-amino antipyrene peroxidase method), serum triglycerides (glycerol phosphatase oxidase − phenol4-amino antipyrene peroxidase method), and high-density lipoprotein (HDL)-cholesterol (cholesterol oxidase phenol4-amino antipyrene peroxidase method) were measured on a spectrophotometer (UV Mini 1240) using Chronolab kits (Chronolab Systems, Barcelona, Spain). Low-density lipoprotein (LDL)-cholesterol was calculated using the Friedwald’s formula [24].

### DNA extraction and molecular genotyping

DNA was extracted from whole blood on filter paper by the Chelex method. Seventy-four participants (37 type 2 diabetic patients and 37 controls) were genotyped for *TCF7L2* rs7903146 by Polymerase Chain Reaction-Restriction Fragment Length Polymorphism (PCR-RFLP). The *TCF7L2* rs7903146 (*C/T*) polymorphism was genotyped using the following primers: Forward 5’-AAG AGA AGA TTC CTT TTT AAA TGG TG-3’, Reverse 5’-CCT CAT ACG GCA ATT AAA TTA TAC A-3’ (SIGMA-ALDRICH, St. Louis, Missouri, United States). A final reaction volume of 15 μL for the Polymerase Chain Reaction (PCR) was constituted, which contained 100 ng of genomic DNA, 5 pmol of each primer, PCR buffer with 1 mmol/L of MgCl_2_, 100 μmol/L of each deoxynucleotide triphosphate (dNTP), 0.5 U of Hot Start *Taq* DNA polymerase (QIAGEN) and 7.8 μl of nuclease free water. The PCR was carried out on a BIOMETRA T3 Thermal Cycler under the following conditions: 95°C for 15 minutes, followed by 34 cycles of 95°C for 30 seconds, 58°C for 30 seconds, 72°C for 30 seconds, and a final extension of 72°C for 9 minutes. The amplicons were analyzed by agarose gel electrophoresis using a 3% agarose gel and positive amplicons digested with *Helicobacter pylori* CH4 III (*Hpy*-CH4III) restriction enzyme at 37°C overnight. The resulting products were separated by electrophoresis on a 2% agarose gel and visualized under a UV transilluminator.

### Sample size calculation

The sample size required to detect the disease variant with an OR of 10 and an expected allele frequency of 0.10 in the general population, with α = 0.05 and β = 0.20 was 21 T2DM patients and 21 healthy controls. The prospective and post-hoc statistical power analyses were calculated using the Power and Sample Size Calculation software and the Quanto statistical program [25].

### Statistical analysis

Allele and genotype frequencies in patients and controls were estimated by direct counting. Qualitative variables were analyzed by chi square (χ^2^) test with Yates’ continuity correction or the Fisher’s exact test when appropriate using Epi Info version 6 (USD, Stone Mountain, USA). Quantitative variables were analyzed by Mann–Whitney *U*-test statistics using the Statistical Package for Social Science (SPSS) version 20.0 for Windows (SPSS, Chicago, Illinois, USA). A *p* value less than 0.05 was considered statistically significant.

## Results

### Characteristics of the study population

As depicted in Table 1, significant differences between T2DM patients and healthy controls were observed for age (median age - 60.00 yrs vs 50.00, *p* < 0.0001), waist-to-hip ratio (median value - 0.97 vs 1.0, *p* < 0.0001), fasting plasma glucose (median level - 1.57 vs 1.00, *p* < 0.0001), total cholesterol (median level - 157.00 vs 197.00, *p* < 0.0001), HDL-cholesterol (median level - 46.00 vs 50.00, *p* < 0.005) and LDL-cholesterol (median level - 82.00 vs 113.00, *p* < 0.0001).

**Table 1:** **Characteristics of the study population**

**Characteristics**	**Type 2 diabetes patients**	**Healthy controls**	***p*** **value**
	**n = 37**	**n = 37**	
**Demographic**			
Male/female (ratio)	17/20 (0.85)	15/22 (0.68)	0.407
Age (years)	60.00 (55.00 – 65.00)	50.00 (45.00 – 54.50)	< 0.0001
**Clinical**			
WHR	0.97 (0.92 – 0.99)	1.0 (1.0 – 1.0)	< 0.0001
SBP (mmHg)	128.00 (115.00 – 150.00)	135.00 (120.50 – 155.50)	0.384
DBP (mmHg)	76.00 (71.50 – 86.50)	84.00 (73.50 – 97.50)	0.068
BMI (kg/m^2^)	28.07 (25.14 – 35.84)	28.00 (26.00 – 32.50)	0.905
**Biological**			
FPG (g/L)	1.57 (1.30 – 2.30)	1.00 (1.00 – 1.00)	< 0.0001
TC (mg/dl)	157.00 (143.00 – 174.00)	197.00 (173.00 – 219.50)	< 0.0001
HDL-C (mg/dl)	46.00 (39.00 – 51.00)	50.00 (45.50 – 56.00)	0.005
LDL-C (mg/dl)	82.00 (74.00 – 96.50)	113.00 (94.00 – 144.00)	< 0.0001
TG (mg/dl)	139.00 (121.00 – 158.00)	143.00 (130.50 – 167.50)	0.191

Data are medians (interquartile range) unless otherwise stated.

WHR: waist-to-hip ratio; SBP: systolic blood pressure; DBP: diastolic blood pressure; BMI: body mass index; FPG: fasting plasma glucose; TC: total cholesterol; HDL: high density lipoprotein cholesterol; LDL: low density lipoprotein cholesterol: TG: triglycerides.

### Genetic variants and type diabetes mellitus

The study population was in Hardy-Weinberg equilibrium (χ^2^ = 0.18, p = 0.67). *TCF7L2* rs7903146 (C/T) was associated with T2DM (*p* = 0.013 for alleles, and *p* = 0.013 for genotypes), Table 2. The minor allele was C and the major allele was T. The C allele was determined to be the risk allele (9.5% patients vs. 0% healthy controls, OR = 16.56), while the T allele was determined to be the protective allele (90.5% patients vs. 100.0% healthy controls, OR = 0.06). The C/C genotype was completely absent in the study population. The C/T genotype was the risk genotype (18.9% patients vs. 0% healthy controls, OR = 18.44), while the T/T genotype was the protective genotype (81.1% patients vs. 100.0% healthy controls, OR = 0.054).

**Table 2:** **Case–control association analysis of** ***TCF7L2*** **rs7903146 (C/T) with type 2 diabetes**

***TCF7L2*** **rs7903146 (C/T)**	**Type 2 diabetic patients**	**Healthy controls**	**OR (95% CI)**	***p*** **value**
**Alleles**				
C	7 (9.5%)	0 (0%)	16.56 (0.92 – 295.41)	0.013
T	67 (90.5%)	74 (100.0%)	1	
Total (2n)	74 (100.0%)	74 (100.0%)		
**Genotypes**				
C/T	7 (18.9%)	0 (0%)	18.44 (1.01 – 335.97)	0.011
T/T	30 (81.1%)	37 (100.0%)	1	
Total (n)	37 (100.0%)	37 (100.0%)		

### Genotype-phenotype correlation

We performed genotype-phenotype correlations to determine if the inaugural disease phenotype varies according to *TCF7L2* genotypes. Since the homozygous C/C genotype was absent in our study population, the protective T/T genotype was modeled relative to the susceptible C/T genotype in all case-only analyses. This case-only analyses were performed according to phenotypic characteristics such as sex, age at diabetes onset, BMI, waist-to-hip ratio, systolic blood pressure and diastolic blood pressure, fasting plasma glucose, total cholesterol, LDL-cholesterol, HDL-cholesterol and triglycerides. No significant genotype-phenotype correlation was observed in the current study, meaning that *TCF7L2* genotypes were not preferentially associated with a specific T2DM phenotype (data not shown).

## Discussion

Despite the growing burden of non-communicable diseases in Africa, especially diabetes, Africans have only participated minimally in genomics research [26]. Indeed, there is dearth information on the genetic variants for T2DM in sub-Saharan African populations. Our study provides first-time insight into the role of the *TCF7L2* rs7903146 (C/T) gene polymorphism in T2DM among Cameroonians. Our findings demonstrate that *TCF7L2* is a susceptibility locus for T2DM and rs7903146 (C/T) a marker for the effect in the Cameroonian population. This finding is consistent with those of other studies in diverse ethnic populations [9-20].

Studies have shown that frequencies of the T allele are highest in Africa, moderate in Asia, and lowest in North-America and Europe [27]. Indeed, as found in a West-African population [18], the T allele was very frequent in our population. Contrary to other studies the T allele was not associated with T2DM, but yielded a protective effect in our population. Less than 10% of individuals carried the risk allele C, while those carrying the homozygous combination were rare. The background frequencies of the *TCF7L2* alleles and genotypes were different between this Cameroonian population and populations of European, Asian and American ancestries [27], indicating genetic heterogeneity across populations and ancestries on this locus. Although the background frequencies varies widely according to ethnicity, *TCF7L2* essentially conveyed similar risk in different populations.

*TCF7L2* was not preferentially associated with a specific disease phenotype. The association of *TCF7L2* with T2DM independently of BMI and waist-to-hip ratio supports the concept of impaired insulin secretion via a deficiency of the TCF proteins which are the gene products of *TCF7L2* [21]. This is consistent with the potential role of the human *TCF7L2* protein in the development of several cell lineages and organs including pancreatic islet development and in adipogenesis, beta-cell survival and insulin secretory granule function, myogenesis and transcriptional regulation of genes for pro-glucagon and glucagon-like peptides (GLP-1 and GLP-2) [22].

Even though the pathophysiology of T2DM remains unclear, there is substantial evidence to suggest that the *TCF7L2* gene strongly predicts the development of TD2M in several ethnic populations and can be considered an important screening tool to identify the population at risk. Therefore, clinical genetic testing for *TCF7L2* should be considered as an actionable indicator for early intervention and prevention of T2DM in Cameroon and sub-Saharan Africa. Also, the confirmation of the association of *TCF7L2* with T2DM in an independent population provides evidence for further consideration of *TCF7L2* and related molecules and pathways as potential therapeutic targets for T2DM.

## Conclusion

*TCF7L2* gene is strongly associated with T2DM in this Cameroonian population. The association is not dependent on a specific T2DM phenotype. Clinical genetic testing for *TCF7L2* can help to predict the occurrence of T2DM in the sub-Saharan African populations.
